# Risk Factors for Implant Failure and Peri‐Implantitis: A Retrospective Cohort Study

**DOI:** 10.1111/clr.70181

**Published:** 2026-09-25

**Authors:** Nathalia Vilela, Bruno C. V. Gurgel, Hélio D. P. da Silva, Claudio M. Pannuti, Poliana M. Duarte

**Affiliations:** ^1^ Department of Periodontology, College of Dentistry University of Florida Gainesville USA; ^2^ Department of Stomatology, Division of Periodontology, School of Dentistry University of São Paulo São Paulo Brazil; ^3^ Department of Dentistry Federal University of Rio Grande do Norte Natal Brazil; ^4^ Department of Dentistry, Dental Research Division Guarulhos University Guarulhos Brazil

**Keywords:** dental implants, implant failure, peri‐implantitis, risk factors

## Abstract

**Objectives:**

This retrospective cohort study evaluated patient‐, implant‐, site‐, procedural‐, biological‐, and prosthesis/mechanics‐related factors associated with early and late implant failure and peri‐implantitis.

**Material and Methods:**

Electronic health record data from a university center were analyzed. Forty patient‐, implant/site‐, biologic‐, and prosthesis‐related variables were evaluated in relation to early failure, late failure, and peri‐implantitis. Bivariate and survival analyses were performed, followed by mixed‐effects regression models appropriate to each outcome. Robustness was assessed via clustered bootstrap resampling, penalized regression, and sensitivity analyses.

**Results:**

A total of 264 patients and 554 implants were assessed. Cumulative implant failure was 6.8% (3.4% early; 3.4% late), and peri‐implantitis incidence was 7.6%. In multivariable analyses, self‐reported penicillin allergy (OR = 7.9; 95% CI: 2.7–23.7) and anterior implant location (OR = 5.4; 95% CI: 2.0–14.0) were associated with higher odds of early implant failure, whereas antihypertensive medication use (OR = 0.26; 95% CI:0.08–0.91) was associated with lower odds (*p* < 0.05). Late implant failure was associated with hypertension (HR = 2.1; 95% CI: 1.3–3.3), previously grafted sites (HR = 1.4; 95% CI:1.0–1.9), and bone‐level implants (HR = 10.4; 95% CI: 5.5–19.5). In cluster‐robust sensitivity analysis, the association between bone‐level implants and late implant failure was attenuated but remained significant (HR = 3.38; 95% CI: 1.96–5.84). Peri‐implantitis was associated with periodontitis (HR = 3.6; 95% CI: 1.89–6.97), excess cement (HR = 2.7; 95% CI: 1.25–5.88), and PLCBL (HR = 2.1; 95% CI: 1.09–4.13) (*p* < 0.05). Robustness analyses generally supported the direction of the identified associations, although some effect estimates were attenuated.

**Conclusions:**

Implant failure and peri‐implantitis are associated with distinct, potentially modifiable patient‐ and treatment‐related factors. Recognition of these factors may help identify patients and implants at increased risk of adverse outcomes.

## 1 Introduction

The use of dental implants to rehabilitate partially and completely edentulous patients has markedly improved functional and aesthetic outcomes. Long‐term success depends on predictable osseointegration, sustained peri‐implant tissue health, and preservation of prosthetic component integrity. Despite the high reported survival rates (Howe et al. 2019; Jung et al. 2012; Kupka et al. 2024), long‐term treatment success is determined by a multifactorial and potentially interacting set of patient‐related, biological, surgical, implant‐related, and prosthetic factors, which may give rise to complications at different stages of treatment. These complications include peri‐implant mucositis, marginal bone loss, peri‐implantitis with progressive bone loss, and mechanical complications of implants or prosthetic components, with implant loss representing the worst‐case outcome. As the number of implants placed worldwide continues to rise (Elani et al. 2018), identifying associated risk and protective factors remains a key area of investigation with direct clinical implications.

Despite advances in implant macro‐ and micro‐design and in surgical techniques to optimize osseointegration, implant loss remains an undesirable complication that incurs additional costs, delays rehabilitation, and adversely affects patients' oral health and well‐being. Early implant loss is defined as failure to achieve osseointegration before prosthetic loading and has been reported to occur in approximately 1.5% of implants and 4% of patients, although these estimates may vary across studies (Tomasi and Derks 2022). Late implant loss is defined as failure to maintain osseointegration after prosthetic connection and functional loading. It has been reported in about 3% of implant sites and 4% of patients over 10‐year observation periods; over 20 years, rates as high as 10% of implants have been described. Although post‐loading failures may also arise from implant or prosthetic component fracture, biomechanical overload, or trauma, peri‐implantitis is widely recognized as the leading cause of late implant failure (Tomasi and Derks 2022).

Peri‐implantitis is a progressive infectious‐inflammatory disease of the peri‐implant soft and hard tissues, arising from an interaction between microbial dysbiosis and the host immune response (Berglundh et al. 2018). It has emerged as a critical determinant of long‐term implant stability and the success of implant‐supported rehabilitation (Monje et al. 2019; Schwarz and Ramanauskaite 2022; Tomasi and Derks 2022). Earlier and updated systematic reviews report subject‐level peri‐implantitis prevalence of approximately 22%, with numerous studies documenting overall prevalence > 10%, particularly with longer time in function (de Vasconcelos Gurgel et al. 2021; Derks and Tomasi 2015; Galarraga‐Vinueza et al. 2025; C.‐T. Lee et al. 2017; Salvi et al. 2019).

Beyond reporting the incidence of implant failure and peri‐implant diseases, many studies have sought to identify risk indicators for these outcomes. Proposed indicators span patient‐, site‐, procedural‐, implant‐, and prosthesis‐related domains, including age and sex; systemic conditions and exposures; medication use; history of periodontitis and adherence to periodontal maintenance; operator experience; three‐dimensional implant positioning; implant location, diameter, length, and manufacturer; placement in regenerated sites; peri‐implant soft‐tissue phenotype; and prosthetic factors such as restoration type, emergence profile, and the precision of the prosthesis–abutment–implant fit and adaptation, among others (de Vasconcelos Gurgel et al. 2021; Galarraga‐Vinueza et al. 2025; Heitz‐Mayfield et al. 2018; Jemt 2017; Kumar et al. 2025; Monje et al. 2019; Tomasi and Derks 2022).

Although several risk indicators for implant failure and peri‐implantitis have been investigated and summarized in systematic reviews and meta‐analyses, the available evidence remains heterogeneous with respect to study populations, outcome definitions, follow‐up periods, and analytical approaches. In addition, some patient‐, implant‐, and treatment‐related variables have received comparatively less attention, and relatively few studies have evaluated a broad range of potential determinants within a single analytical framework. Defining factors that increase the risk of implant failure and peri‐implantitis is essential for treatment planning, risk stratification, and prevention. Therefore, the aim of this retrospective cohort study was to comprehensively assess patient‐, implant‐, site‐, procedural‐, biological‐, and prosthesis/mechanics‐related factors associated with the incidence of early implant failure, late implant failure, and peri‐implantitis. By evaluating both established and less‐studied variables within a single cohort, this study seeks to contribute additional evidence regarding factors associated with implant outcomes and to identify variables that warrant further investigation.

## 2 Material and Methods

### 2.1 Study Population

This single‐center, university‐based retrospective cohort study used electronic health record (EHR) data from patients who received at least one dental implant at the University of Florida College of Dentistry (Gainesville, Florida, USA) between January 2011 and June 2021. The study protocol was approved by the University of Florida Institutional Review Board (IRB# 202101382). The conduct and reporting of the study followed the Strengthening the Reporting of Observational Studies in Epidemiology (STROBE) guidelines.

### 2.2 Inclusion Criteria

Patients who received at least one dental implant at the University of Florida Dental Clinics were eligible for inclusion. For assessment of early failure, all implants were eligible if they had clinical and radiographic documentation from implant placement through uncovering, abutment installation, or prosthesis delivery. Implants without early failure were considered eligible for evaluation of late failure and peri‐implantitis if they had received a definitive prosthesis and had been in functional occlusion for a minimum of 12 months.

### 2.3 Exclusion Criteria

Patient and implant exclusion criteria included incomplete or unavailable information required for the collection of patient‐, implant/site‐, and prosthesis‐related predictor variables or for outcome assessment, insufficient follow‐up, and inaccessible records. Implants were also excluded when the required radiographic assessment time points were missing, or when inadequate radiographic quality (e.g., distortion or superimposition) precluded reliable evaluation of peri‐implant bone levels for pre‐loading crestal bone loss (PLCBL) and peri‐implantitis assessment.

### 2.4 Data Collection

#### 2.4.1 Variables

Information regarding patients and implants was manually extracted from an EHR database (AxiUm, Las Vegas, NV, USA) by two trained examiners (B.C.V.G. and N.V.) between September 2021 and July 2023. The examiners performed a detailed review of completed treatment codes, clinical notes, and attachments containing biomaterial specifications and radiographs. All collected data were entered into an extraction form specifically created for this study.

Patient‐related information included: age at the time of implant placement (years); sex (male/female); smoking status (current, former, never); daily alcohol consumption; systemic diseases/conditions (hypertension, diabetes mellitus, hypothyroidism, osteoporosis, and/or self‐reported penicillin allergy); medications (antihypertensive, anti‐inflammatory, antidepressant and/or antiresorptive [denosumab or bisphosphonates]); history of periodontitis (yes or no); frequency of supportive periodontal therapy (≤ 1 time/year, 2–3 times/year, or ≥ 3 times/year); and periodontal status. Periodontal status was categorized as periodontitis or no periodontitis. Periodontitis was defined as the presence of ≥ 2 interdental sites with clinical attachment loss (CAL) ≥ 1 mm, probing depth (PD) ≥ 4 mm with bleeding on probing (BoP) (Papapanou et al. 2018; Tonetti et al. 2018). “No periodontitis” was defined as the presence of periodontal health or gingival inflammation on an intact or reduced periodontium in a non‐periodontitis patient, as well as a successfully treated and clinically stable periodontitis patient, consistent with the 2017 World Workshop classification of periodontal and peri‐implant diseases and conditions (Chapple et al. 2018).

Implant/site‐related data included: location (mandible or maxilla; anterior or posterior); implant manufacture (Straumann, Astra, Zimmer, or other manufacturers); diameter (narrow ≤ 3.6 mm, regular > 3.6–≤ 4.8 mm, or wide > 4.8 mm); length (short ≤ 8 mm or conventional > 8 mm); implant platform–abutment interface (bone‐level or tissue‐level); healing mode (submerged/two‐stage or non‐submerged/one‐stage); timing of placement (immediate or delayed); implant‐tooth/implant–implant distance (inadequate [< 1.5 mm/< 3 mm] or adequate [≥ 1.5 mm/≥ 3 mm]); insertion depth (equicrestal, subcrestal, or supracrestal); operator (faculty or resident); bone graft during implant placement (yes or no); previous bone grafting (none, ridge preservation, guided bone regeneration [GBR], lateral or crestal sinus lift); and immediate provisionalization.

Prosthesis‐related data included: type of restoration (single or bridge/splinted); crown retention (screw‐retained or cemented); crown contour (adequate or overcontoured; assessed via radiographs); marginal adaptation (adequate or defective; assessed via radiographs); interproximal contact (adequate or open contact); excess cement (present or absent, assessed via radiographs); technical issues (none, chipping, decementation, screw loosening, or abutment loosening); implant/prosthesis component fracture (yes or no); and antagonist (natural teeth or prosthesis).

Biologic variables related to late failure and peri‐implantitis included PLCBL, defined as a reduction of ≥ 0.5 mm in interproximal bone level from implant placement to uncovery or abutment installation or crown delivery. Peri‐implantitis, as defined below, was also included as a biologic variable associated with late implant failure.

#### 2.4.2 Outcomes

##### 2.4.2.1 Implant Failure

Early implant failure was defined as implant loss occurring before loading due to a lack of osseointegration. Late implant failure was defined as implant loss occurring after successful osseointegration and loading with the final restoration (Esposito et al. 1998).

##### 2.4.2.2 Peri‐Implantitis

Peri‐implantitis was diagnosed when all of the following criteria were met: bleeding and/or suppuration on probing; increased probing depth (PD) compared with baseline examinations and/or PD ≥ 6 mm; and radiographic evidence of progressive bone loss beyond initial crestal bone remodeling (Berglundh et al. 2018). Based on the recommendations of Renvert et al. (2018), radiographic bone loss was defined as bone loss of ≥ 2 mm apical to the most coronal portion of the intraosseous implant. This threshold was selected to improve diagnostic specificity and minimize potential misclassification resulting from physiological crestal bone remodeling following implant placement and loading, as well as radiographic measurement variability. Furthermore, a peri‐implantitis diagnosis was considered only when these criteria were met at least one year after delivery of the implant‐supported prosthesis (Berglundh et al. 2018; Renvert et al. 2018).

### 2.5 Radiographic Assessment

Due to the retrospective design, strict radiographic standardization could not be ensured for all implants. Radiographic assessment for PLCBL and peri‐implantitis diagnosis was performed using available digital periapical or bitewing radiographs obtained as part of routine clinical care using film holders.

PLCBL was defined as a bone level change of ≥ 0.5 mm from implant placement to implant uncovering, abutment installation, or prosthesis delivery. For peri‐implantitis, radiographic bone loss was defined as a bone level change of ≥ 2.0 mm between the radiograph obtained at prosthesis delivery and the radiograph obtained at the visit when peri‐implantitis was identified.

Measurements were performed on the mesial and distal aspects of each implant by a single trained and calibrated examiner (N.V.) using a calibrated linear measurement tool (MiPACS, Charlotte, NC, USA), with the known implant diameter or length used for image calibration, as previously described (Vilela et al. 2024). Intra‐examiner measurement reproducibility was excellent, as demonstrated by a concordance correlation coefficient (CCC) of 0.997.

### 2.6 Statistical Analyses

Descriptive statistics were used to summarize patient‐, implant/site‐, prosthesis‐, and biological variables, including their distributions, frequencies, means, and standard deviations. Missing data were handled using a complete‐case approach. Patient or implants were excluded if information required for the eligibility assessment, outcome definition, or variables included in the analyses was missing or unavailable. No data imputation was performed. No a priori sample size calculation was performed, as all implants meeting the inclusion and exclusion criteria during the study period were included to maximize representativeness and statistical precision.

The Pearson's Chi‐squared test or Fisher's Exact test was applied, as appropriate, based on expected cell counts, for bivariate comparisons of study variables according to the occurrence of early and late implant failure and peri‐implantitis. Bivariate analyses were used both to describe associations between clinical variables and each outcome and to identify potential predictors for multivariable modeling. Variables with *p* < 0.10 in bivariate analyses were subsequently entered into multivariable mixed‐effects models, accounting for implant clustering within patients.

Mixed‐effects logistic regression models were applied for early implant failure (a binary outcome without a time dimension) to estimate odds ratios (ORs) and corresponding 95% confidence intervals (CIs). For late implant failure and peri‐implantitis, mixed‐effects Cox proportional hazards models were used to account for time‐to‐event data and to estimate hazard ratios (HRs) and 95% CIs. For these time‐to‐event analyses, the time origin was defined as the date of prosthesis delivery and implants were followed until the occurrence of the event of interest or censoring at the last recorded follow‐up.

A backward stepwise elimination procedure was applied to identify parsimonious multivariable models. Variables with *p* < 0.05 in the final models were considered statistically significant. Model fit and model comparisons were assessed using likelihood ratio tests, when applicable, and further evaluated using the Akaike Information Criterion (AIC), Bayesian Information Criterion (BIC), and pseudo‐*R* ^2^ values. Because implant failure is a relatively rare outcome, the number of observed failures remained limited despite the inclusion of 554 implants. Therefore, events‐per‐variable (EPV) ratios were assessed for all final multivariable models to evaluate the potential for model instability and overfitting. To further examine the robustness of the findings, several complementary analyses were performed, including patient‐level clustered bootstrap resampling (2000 iterations), penalized regression methods (least absolute shrinkage and selection operator [LASSO] and Elastic Net), and assessment of multicollinearity using variance inflation factors (VIFs). Sensitivity analyses restricted to implants with a minimum post‐loading follow‐up period were also conducted for the peri‐implantitis outcome. Detailed EPV assessments, multicollinearity diagnostics, and additional robustness analyses are provided in the Supporting Information and Supporting Tables, including Tables S1–S6 and Figures S1–S5.

Survival curves for late implant failure and peri‐implantitis were generated using the Kaplan–Meier method for variables that showed significant differences according to the log‐rank (Mantel–Cox) test. The significance level was set at 5% for all analyses.

## 3 Results

The EHR database identified 3336 patients who received at least one dental implant between January 2011 and June 2021. After removing duplicate records, 536 were randomly selected for detailed review using a random number generator from September 2021 to July 2023. Manual evaluation of clinical notes and radiographic records resulted in the exclusion of 272 cases based on predefined exclusion criteria. The final study sample comprised 264 patients and 554 dental implants included in the analysis (Figure 1). All 554 implants were assessed for early implant failure, 535 implants for late implant failure, and 534 implants for peri‐implantitis. The overall cumulative failure rate at implant was 6.8%, comprising 3.4% early failures and 3.4% late failures. In addition, 7.6% of implants developed peri‐implantitis.

**FIGURE 1:**
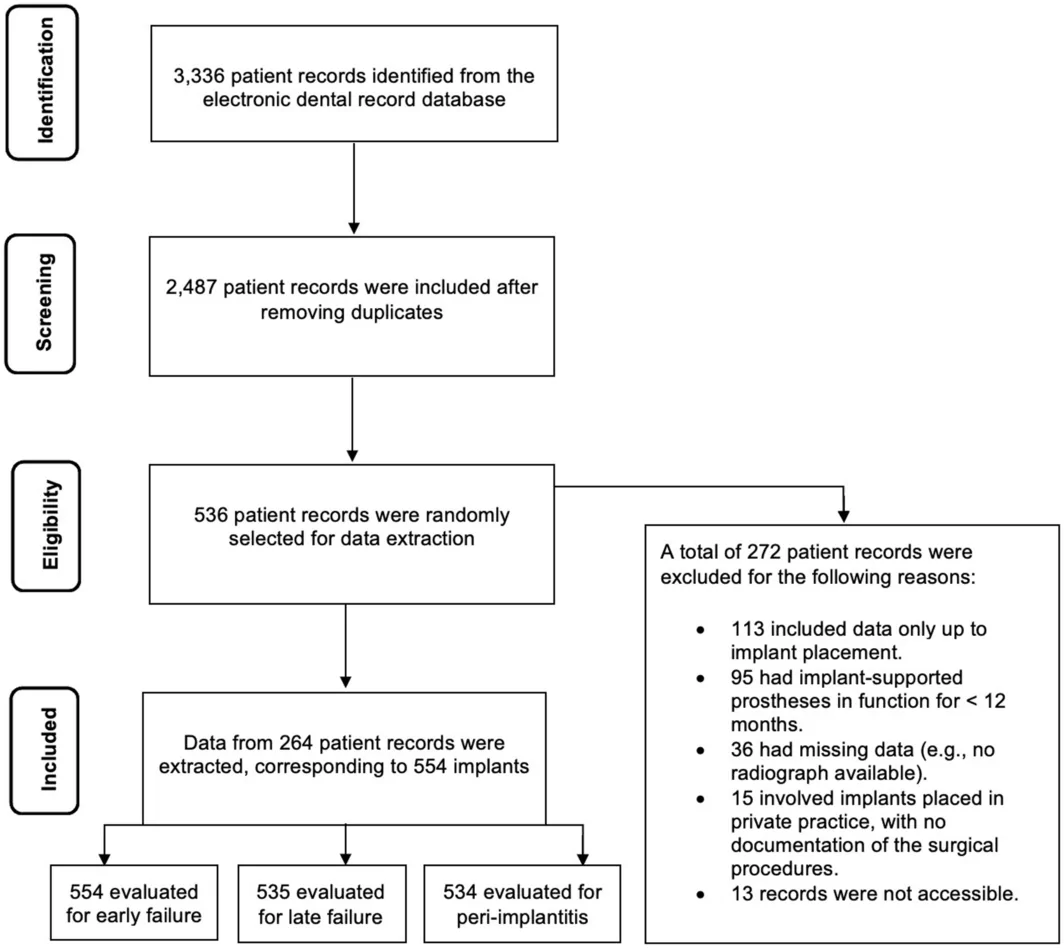
Study flowchart.

The mean age of the study population at the time of implant placement was 60 ± 12.2 years, and the mean post‐loading follow‐up time was 5.0 ± 3.0 years (range: 1–12 years).

Unadjusted bivariate analyses of associations between patient‐, implant/site‐, prosthesis‐, and biologic‐related variables and early implant failure, late implant failure, and peri‐implantitis are presented in supporting Tables S1–S4, respectively. Detailed descriptions of Tables S1–S4 are provided in the supporting information (Data S1).

Figure 2 displays the Kaplan–Meier cumulative survival curves for variables significantly associated with late failure. Significantly lower late implant survival was observed in hypertensive and self‐reported penicillin‐allergic patients compared with their counterparts (*p* = 0.012 and *p* = 0.018, respectively). Straumann implants showed the highest late survival probability (99.1%), followed by Astra (96.1%) and Zimmer (92.7%) (*p* = 0.033). Implants placed in areas treated with GBR or sinus lift procedures exhibited lower survival probabilities (87.1% and 89.5%, respectively) than those placed in native bone or in sites that underwent ridge preservation (97.8% and 97.2%, respectively; *p* = 0.003). Tissue‐level implants demonstrated significantly higher survival than bone‐level implants (99.4% versus 95.4%; *p* = 0.001). Implants with implant/prosthesis component fracture or PLCBL had significantly lower survival than their counterparts (*p* < 0.001 and *p* = 0.001, respectively). Implants affected by peri‐implantitis exhibited markedly reduced survival compared with those without the condition (*p* = 0.00), with a mean survival time of 8.5 ± 0.6 years and 75% survival reached at 4.7 years. Although overall late implant survival remained high, failures tended to occur earlier during follow‐up in implants exposed to the abovementioned conditions compared with non‐exposed implants.

**FIGURE 2:**
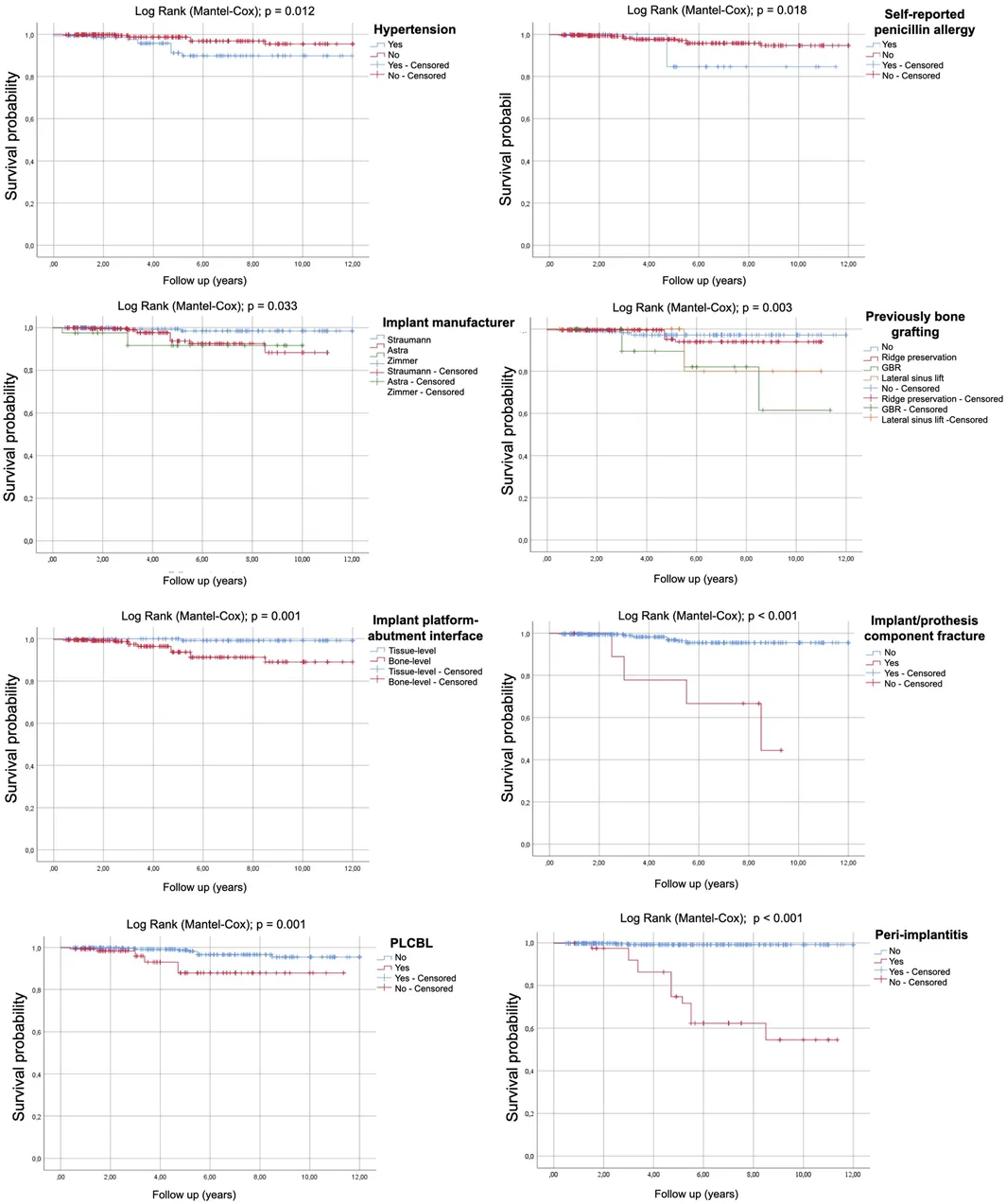
Kaplan–Meier survival curves for late implant failure according to variables significant in the Log‐Rank (Mantel–Cox) test (*p* < 0.05): Hypertension, self‐reported penicillin allergy, implant manufacturer, previous bone grafting, implant platform‐abutment interface, implant/prosthesis component fracture, PCBL (pre‐loading crestal bone loss), and peri‐implantitis.

Figure 3 displays the Kaplan–Meier cumulative survival curves for variables significantly associated with peri‐implantitis‐free survival. The probability of remaining free from peri‐implantitis was significantly lower for patients with hypothyroidism and periodontitis than for those without these conditions (*p* < 0.001). Implants placed by residents exhibited significantly lower peri‐implantitis‐free survival compared with those placed by faculty members (*p* = 0.001). Straumann implants exhibited the highest peri‐implantitis‐free survival, followed by Astra and Zimmer (*p* = 0.036). Implants placed in previously augmented sites, particularly those treated with GBR or sinus lift, showed a lower probability of remaining free from peri‐implantitis compared with those placed in native bone or after ridge preservation (*p* < 0.001). In addition, implants with excess cement or PLCBL demonstrated significantly lower peri‐implantitis‐free survival than their counterparts (*p* = 0.009 and *p* = 0.017, respectively). Despite overall high peri‐implantitis–free survival, implants exposed to the factors identified in this analysis showed earlier onset and a higher cumulative incidence of peri‐implantitis during follow‐up (Figure 3).

**FIGURE 3:**
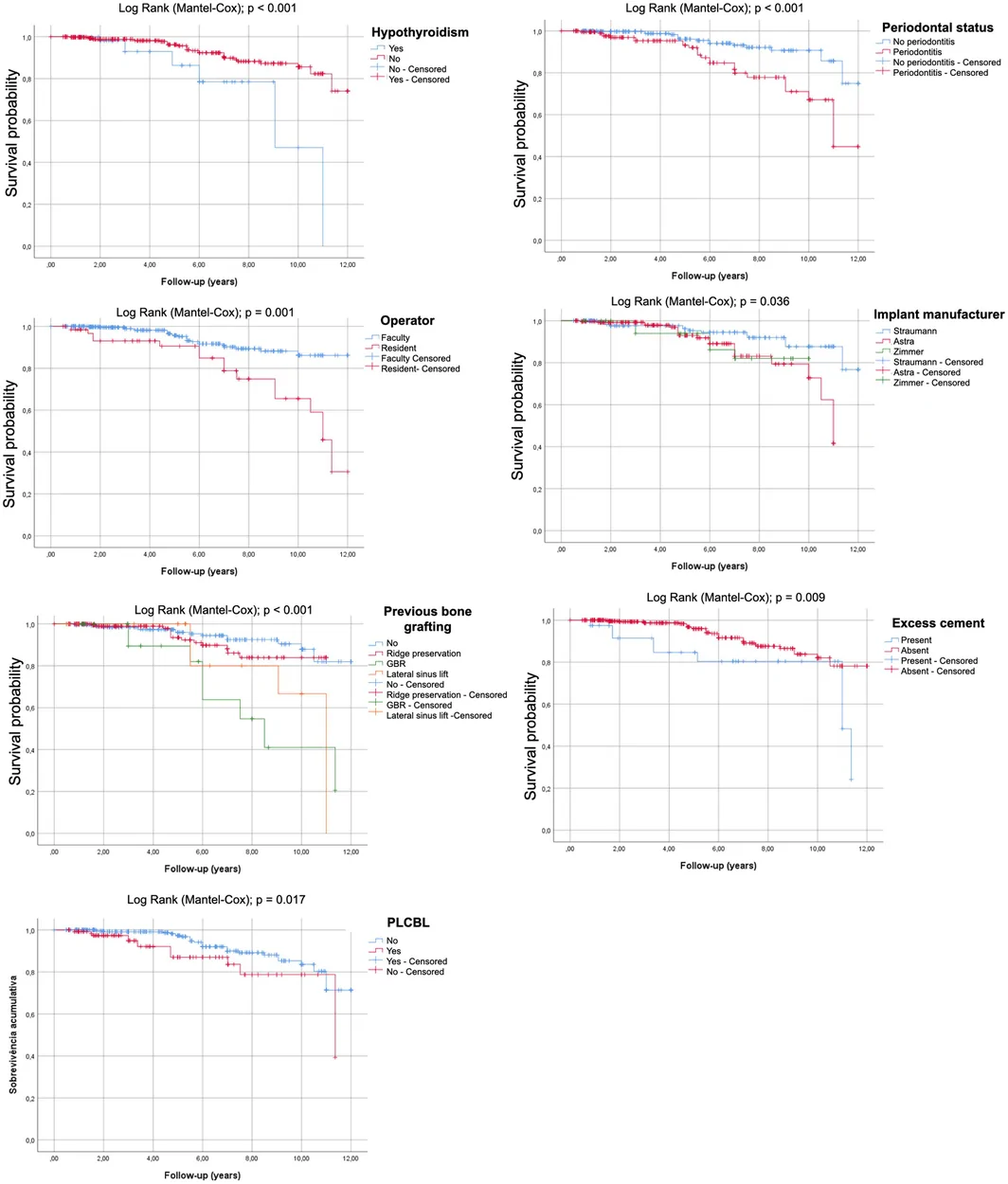
Kaplan–Meier survival curves for peri‐implantitis according to variables significant in the Log‐Rank (Mantel–Cox) test (*p* < 0.05): Hypothyroidism, periodontal status, operator, implant manufacturer, previous bone grafting, excess cement, PCBL (pre‐loading crestal bone loss).

According to the exploratory mixed‐effects logistic regression model, patients with a self‐reported penicillin allergy had a higher likelihood of early implant failure compared with non‐allergic patients (OR = 7.9, 95% CI: 2.7–23.7; *p* < 0.001). Implants placed in posterior regions presented a lower risk of early failure than those in anterior sites (OR = 0.19, 95% CI: 0.07–0.5; *p* < 0.001). Furthermore, patients using antihypertensive medications had lower odds of early implant failure compared with those not receiving such medication (OR = 0.26, 95% CI: 0.08–0.91; *p* = 0.029) (Table 1).

**TABLE 1:** Final mixed‐effects logistic regression model for early implant failure.

Variable	Contrast	*β* (SE)	OR	95% CI	*z*	*p*
Allergy to penicillin	Yes vs. no	2.1 (0.6)	7.9	2.7–23.7	3.7	< 0.001
Antihypertensive medications	Yes vs. no	−1.3 (0.6)	0.26	0.08–0.91	−2.2	0.029
Implant location	Posterior vs. anterior	−1.7 (0.5)	0.19	0.07–0.50	3.4	< 0.001

Abbreviations: CI, 95% confidence interval; OR, odds ratio.

A comparison of OR obtained from the exploratory mixed‐effects logistic regression model and the patient‐level clustered bootstrap analysis for early implant failure is presented in Figure S1. Although the direction and overall magnitude of the associations remained consistent across analytical approaches, confidence intervals widened substantially in the clustered bootstrap analysis, reflecting the limited number of failure events and reduced precision of the effect estimates. Results of the LASSO analysis for early implant failure are presented in the Supporting Information, Table S5 and Figure S2.

According to the mixed‐effects Cox proportional hazards model, hypertensive patients showed a higher hazard of late implant failure compared with non‐hypertensive individuals (HR = 2.1, 95% CI: 1.3–3.3; *p* = 0.004). Implants placed in sites also exhibited an increased risk (HR = 1.4, 95% CI: 1.0–1.9; *p* = 0.041). Bone‐level implants demonstrated a greater hazard of late failure than tissue‐level implants (HR = 10.4, 95% CI: 5.5–19.5; *p* < 0.001). Additionally, Astra implants presented a higher hazard of late failure compared with Straumann implants (HR = 2.4, 95% CI: 1.3–4.4; *p* = 0.006) (Table 2).

**TABLE 2:** Multivariable Cox proportional hazards analyses for late implant failure, including primary and sensitivity analyses.

Variable	Contrast	Primary mixed‐effects Cox model					Sensitivity analyses	
		*β* (SE)	HR	95% CI	*z*	*p*	Cluster‐robust HR (95% CI)	Clustered bootstrap HR (95% CI)
Hypertension	Yes vs. no	0.7 (0.3)	2.1	1.3–3.3	2.9	0.004	1.41 (1.06–1.88)	1.41 (1.05–1.92)
Previous bone grafting	Yes vs. no	0.3 (0.2)	1.4	1.0–1.9	2.0	0.041	1.20 (0.93–1.54)	1.20 (0.92–1.59)
Implant platform‐abutment interface	Bone vs. tissue level	2.3 (0.3)	10.4	5.5–19.5	7.3	0.000	3.38 (1.96–5.84)	3.38 (2.12–7.04)
Implant manufacturer	Astra vs. Straumann	0.9 (0.3)	2.4	1.3–4.4	2.8	0.006	1.67 (0.95–2.93)	1.67 (0.98–3.25)

The results of the patient‐level clustered bootstrap resampling analysis (2000 iterations) for late implant failure are presented in Table 2 and Figure S3, together with a comparison of effect estimates and 95% confidence intervals from the primary mixed‐effects Cox proportional hazards model and a cluster‐robust Cox proportional hazards regression model. Although the magnitude of the effect estimates varied across analytical approaches, the direction of the associations remained consistent. The greatest attenuation was observed for the association between bone‐level implants and late implant failure, with the hazard ratio decreasing from 10.4 (95% CI: 5.45–19.5) in the primary mixed‐effects Cox model to 3.38 (95% CI: 1.96–5.84) in the cluster‐robust analysis and 3.38 (95% CI: 2.12–7.04) in the clustered bootstrap analysis. These findings support the overall robustness of the identified predictors while highlighting some uncertainty regarding the magnitude of the observed effects. Detailed results of the LASSO analysis for late implant failure are presented in the Supporting Information and Figure S4.

The mixed‐effects Cox proportional hazards model showed that patients with periodontitis had a significantly higher hazard of developing peri‐implantitis compared with those without periodontitis (HR = 3.6, 95% CI: 1.89–6.97, *p* < 0.001). Implants with excess cement also exhibited a greater hazard of peri‐implantitis than those without cement remnants (HR = 2.7, 95% CI: 1.25–5.88, *p* = 0.012). Furthermore, implants that presented PLCBL were at increased risk of peri‐implantitis compared with those without PLCL (HR = 2.1, 95% CI: 1.09–4.13, *p* = 0.027) (Table 3).

**TABLE 3:** Final mixed‐effects Cox proportional hazards model for peri‐implantitis.

Variable	Contrast	*β* (SE)	HR	95% CI	*z*	*p*
Periodontal status	Periodontitis vs. no periodontitis	1.29 (0.33)	3.6	1.89–6.97	15.04	< 0.001
Excess cement	Present vs. absent	0.99 (0.40)	2.7	1.25–5.88	6.36	0.012
PLCBL	Yes vs. no	0.75 (0.34)	2.1	1.09–4.13	4.87	0.027

Abbreviations: CI, 95% confidence interval; HR, hazard ratio; PLCBL, pre‐loading crestal bone loss.

Sensitivity analyses restricted to implants with at least 3 and 5 years of post‐loading follow‐up are presented in Figure 4 and Table 4. As expected, restricting the cohort reduced both the sample size and the number of peri‐implantitis events (41 events in the primary analysis, 35 events in the ≥ 3‐year cohort, and 24 events in the ≥ 5‐year cohort), resulting in wider confidence intervals and reduced statistical precision. Overall, most associations maintained the same direction across the sensitivity analyses, supporting the robustness of the primary findings. The association between periodontitis status and peri‐implantitis remained directionally consistent across all analyses and remained significant in the primary and in the ≥ 5‐year models, whereas the associations for excess cement and PLCBL were attenuated after restricting the cohort to implants with longer follow‐up periods.

**FIGURE 4:**
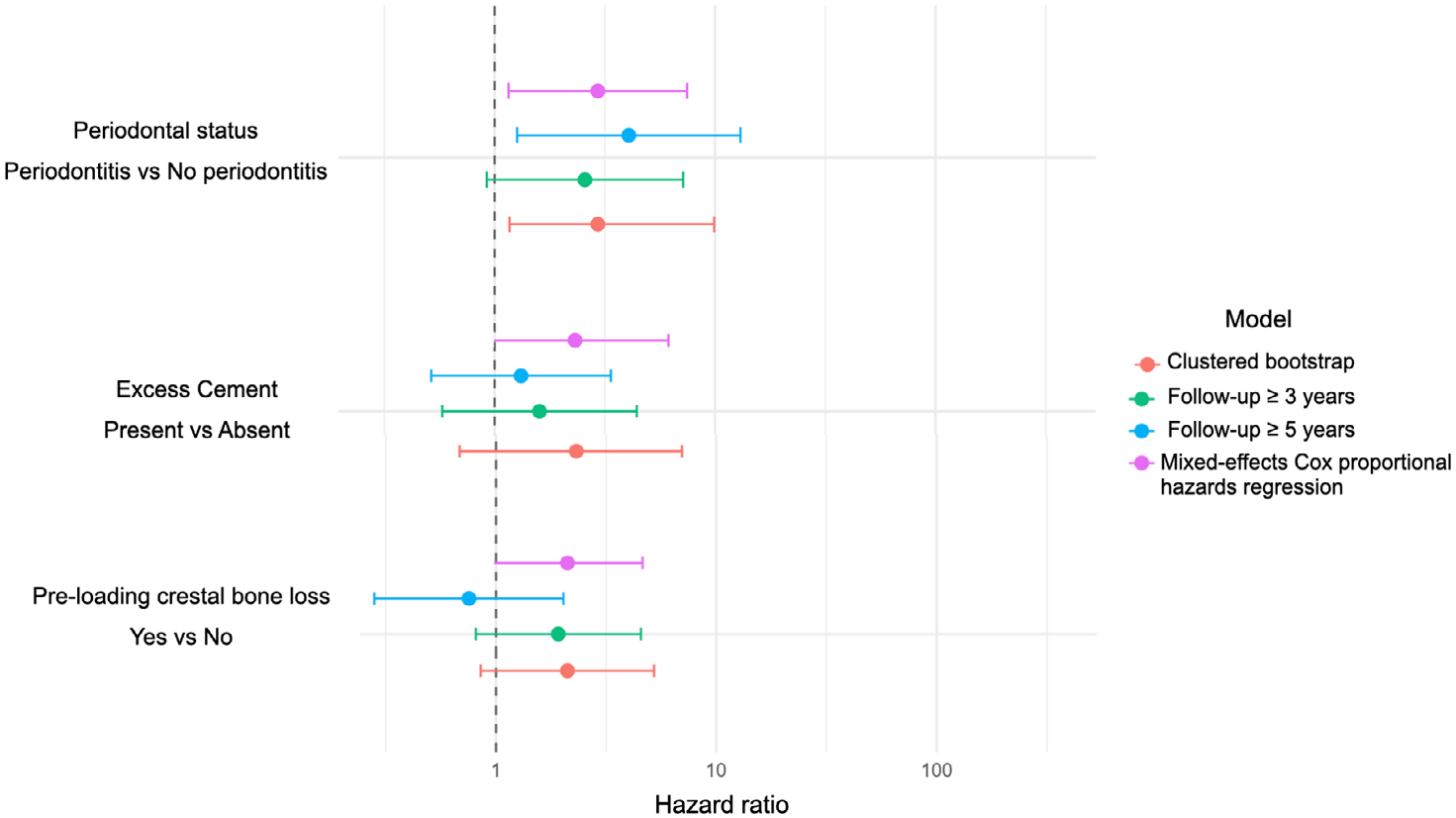
Comparison of effect estimates and 95% confidence intervals for peri‐implantitis obtained from the primary mixed‐effects Cox proportional hazards model, patient‐level clustered bootstrap resampling (2000 iterations), and sensitivity analyses restricted to implants with at least 3 and 5 years of follow‐up.

**TABLE 4:** Sensitivity analyses restricted to implants with longer post‐loading follow‐up.

Analysis	Implants (*n*)	Events (*n*)	Periodontitis status HR (*p*)	Excess cement HR (*p*)	PLCBL HR (*p*)
Primary model	534	41	3.63 (< 0.001)	2.70 (0.012)	2.12 (0.027)
Follow‐up ≥ 3 years	365	35	2.57 (0.071)	1.60 (0.365)	1.92 (0.138)
Follow‐up ≥ 5 years	257	24	4.06 (0.018)	1.32 (0.563)	0.75 (0.577)

Abbreviations: HR, hazard ratio; PLCBL, pre‐loading crestal bone loss.

## 4 Discussion

This study evaluated associations between multiple patient‐, implant/site‐, prosthesis‐, and biology‐related variables and early and late implant failure, as well as peri‐implantitis. Self‐reported penicillin allergy and anterior implant location were associated with increased odds of early implant failure, whereas antihypertensive medication use demonstrated a protective effect. Hypertension, implant placement in previously grafted sites, bone‐level platform placement, and implant manufacturer were associated with an increased likelihood of late implant failure. For peri‐implantitis, current periodontitis, PLCBL, and excess cement emerged as the main risk indicators. Overall, the complementary robustness analyses supported the direction of the observed associations. Although some variability in effect magnitude and predictor selection was observed for the implant failure outcomes, the main findings were generally preserved across analytical approaches. Given the limited number of implant failure events, the early and late failure results should be interpreted as exploratory and hypothesis‐generating. For peri‐implantitis, the associations identified in the primary analysis were generally maintained across the complementary analyses, providing additional support for the observed findings.

### 4.1 Early Implant Failure

Early implant failure occurred at a rate of 3.4% at the implant level, consistent with low rates reported in the literature (Jemt et al. 2016; Lang et al. 2025; van Steenberghe et al. 2002; Yari et al. 2024).

Penicillin allergy is commonly reported worldwide, with an estimated prevalence of nearly 10% (Luintel et al. 2025), although most self‐reported cases are not confirmed by formal allergy testing (Lozo et al. 2023). Self‐reported penicillin allergy and the use of alternative antibiotics have been associated with higher postoperative complication rates across various oral surgery procedures (Basma and Misch 2021; Boussaïd et al. 2024; Roistacher et al. 2022). In the present study, self‐reported penicillin allergy was associated with an increased risk of early implant failure, consistent with previous studies and systematic reviews reporting higher implant failure rates among individuals who did not receive perioperative penicillin or who received alternative antibiotic regimens. (Block et al. 2021; Edibam et al. 2023; French et al. 2015, 2016; Zahra et al. 2022). Although the mechanisms underlying this association remain unclear, self‐reported penicillin allergy and the consequent avoidance of perioperative penicillin may influence implant outcomes through altered infection control or differences in the effectiveness of alternative antibiotic regimens. Given that most reported penicillin allergies are not confirmed by formal testing, allergy assessment and delabeling may have important implications for implant therapy. Nevertheless, the present findings should be interpreted cautiously, and larger studies are needed to confirm this association and better estimate its magnitude.

Implants placed in anterior regions showed a higher risk of early failure, consistent with previous reports of greater failure rates in anterior compared with posterior sites (Fouda 2020; Lázaro‐Abdulkarim et al. 2022; Lin et al. 2018; Silva et al. 2025). This association may reflect anatomical and procedural factors typical of the anterior zone, such as thinner alveolar ridges and the more frequent use of immediate placement, immediate provisionalization, and narrow‐diameter implants. In line with this, both immediate provisionalization and narrow‐diameter implants were significantly associated with early failure in the bivariate analysis (Table S2).

Self‐reported use of antihypertensive medications was identified as a protective factor against early implant failure in the primary analysis. However, this association was not consistently retained across all robustness analyses and should therefore be interpreted with caution. Our exploratory findings are consistent with previous studies reporting lower implant failure rates among patients using antihypertensive medications (Masri et al. 2025; Wu et al. 2016). Although the biological basis of this protective effect remains incompletely understood, preclinical and clinical evidence suggest that certain antihypertensive agents may positively influence bone metabolism and healing (Graham et al. 2008; Wu et al. 2016; Zhang et al. 2023). Because antihypertensives were analyzed as a single category, future studies should evaluate the effects of specific drug classes, such as beta‐blockers, thiazide diuretics, and angiotensin II receptor antagonists, on osseointegration and implant failure.

### 4.2 Late Implant Failure

Late implant failure occurred in 3.4% of implants in this cohort, a rate comparable to those reported in previous studies (Derks and Tomasi 2015; Friberg and Jemt 2015; Lang et al. 2025).

Hypertension has been evaluated as a potential risk factor for implant failure in few studies, most of which reported no significant association (Hamadé et al. 2024; Moy et al. 2005; Tonini et al. 2022). In contrast, our study identified hypertension as a significant patient‐related risk factor for late implant failure across bivariate (Table S1), survival (Figure 2), and multivariable (Table 2) analyses, even after adjustment for confounders. The association remained directionally consistent across the robustness analyses (Figure S3), supporting the stability of this finding. Differences from prior reports may reflect variations in sample size, study design, statistical modeling, and reliance on self‐reported hypertension. Although still understudied in implant research, hypertension has been associated with impaired bone metabolism and reduced bone mineral density (Gao et al. 2025; Ye et al. 2017). Through activation of the renin–angiotensin–aldosterone system and modulation of the osteoprotegerin/RANKL pathway, hypertension may promote bone loss (Mo et al. 2020) and increase susceptibility to late implant failure. However, additional clinical and mechanistic studies are needed to confirm and further elucidate this relationship.

Implants placed in previously grafted sites were associated with an increased risk of late implant failure (Table 2). This association remained consistent across robustness analyses (Figure S3), although variability in confidence intervals suggests uncertainty regarding the precision of the estimate. Previous studies have generally reported high implant survival and comparable outcomes between grafted and non‐grafted sites (Buser et al. 2002; Clementini et al. 2012; Gurbanov and Plugmann 2024; Hämmerle et al. 2002; Meijndert et al. 2008; Salvi et al. 2018; Tran et al. 2016; Urban et al. 2009). However, augmentation procedures of varying complexity are often analyzed together, and implant failures are typically reported as a single outcome. In the present study, the association observed for previously grafted sites appeared to be driven primarily by implants placed in sites treated with GBR, which demonstrated a higher incidence of late failure and an earlier onset of failure (Table S2, Figure 2). This finding is consistent with studies identifying GBR as a potential risk factor for implant failure (French et al. 2015; Mayta‐Tovalino et al. 2019), although conflicting findings have also been reported (Hong et al. 2020; Tatli et al. 2025; Zumstein et al. 2019). Notably, implants placed in sinus lift sites showed a similar trend toward reduced survival (Figure 2); however, this association did not reach statistical significance in the bivariate analysis and should be interpreted with caution, particularly given the smaller sample size. These findings suggest that the increased risk observed in previously grafted sites may not be uniform across augmentation procedures. Further research is needed to determine whether the observed risk is primarily related to specific augmentation techniques, biological characteristics of the regenerated tissue (e.g., residual graft particles or altered bone remodeling), reduced native bone availability, or biomechanical factors associated with more extensive reconstructions, such as increased functional demands and unfavorable crown‐to‐implant ratios.

Bone‐level implants were associated with a higher risk of late implant failure in the primary analyses (Table S2, Figure 2 and Table 2); however, this finding should be interpreted with caution. The magnitude of the association was attenuated in the robustness analyses (Figure S3), although its direction remained consistent across analytical approaches. These findings support a potential post‐loading protective effect of positioning the implant–abutment interface coronal to the crestal bone, a characteristic feature of tissue‐level implants. Although previous systematic reviews reported similar survival and peri‐implantitis risk between tissue‐level and bone‐level implants within 1–5 years of follow‐up (Atieh et al. 2025; Cosola et al. 2020; Vouros et al. 2012), the longer follow‐up period in the present study suggests that the influence of implant–abutment interface position on implant outcomes may become more apparent over time. Importantly, previous investigations have shown that tissue‐level implants are associated with lower pre‐loading and post‐loading marginal bone loss (Agustín‐Panadero et al. 2021; van Eekeren et al. 2016; Vilela et al. 2024). In the present study, PLCBL was associated with peri‐implantitis (Table S4; Table 3; Figure 3), increased late implant failure, and reduced survival probability (Table S4; Figure 2). Collectively, these findings support a potential relationship between implant–abutment interface position, crestal bone stability, peri‐implantitis, and late implant failure, suggesting a long‐term advantage of tissue‐level implants. While differences in the location of the implant–abutment interface may contribute to distinct biological and mechanical responses, residual confounding and selection bias inherent to the retrospective design cannot be excluded. Factors such as soft‐tissue phenotype, bone volume and anatomical complexity may have influenced implant selection and contributed to the more favorable outcomes observed for tissue‐level implants. Therefore, the observed association should not be interpreted as evidence of a causal effect of implant platform design alone. Finally, the lower rate of late failure observed for Straumann implants compared with Astra implants (Table 2) may be partly explained by differences in platform‐abutment interface, as all Astra implants were bone‐level, whereas Straumann implants included both bone‐level and tissue‐level designs. Given this structural overlap between manufacturer and platform‐abutment interface, their individual effects cannot be considered fully independent and should therefore be interpreted with caution. Nevertheless, other manufacturer‐related factors, including differences in implant macro‐ and micro‐design, surface characteristics, and surgical protocols, may also have contributed to this finding and warrant further investigation.

Self‐reported penicillin allergy, screw or prosthetic component fracture, PLCBL, and peri‐implantitis were not retained in the final model but showed significant associations with late failure in bivariate and survival analyses. These findings suggest that late implant failure may be biologically linked to PLCBL and peri‐implantitis, reflecting a sequence of early marginal bone disruption, bacterial colonization, and progressive inflammatory breakdown leading to implant loss. Similarly, screw or component fracture may contribute to late failure through mechanical compromise of implant integrity. Self‐reported penicillin allergy has also been associated with PLCBL in previous studies (Vilela et al. 2024). Nevertheless, whether this association contributes to late implant failure remains unclear and warrants further investigation.

### 4.3 Peri‐Implantitis

Consistent with our findings, several studies have demonstrated a clear association between peri‐implantitis and increased implant failure over time (Daubert et al. 2015; Jemt and Eriksson 2020; D. W. Lee 2014; Sgolastra et al. 2015). Using the diagnostic criteria established by the 2017 World Workshop on the Classification of Periodontal and Peri‐Implant Diseases and Conditions (Berglundh et al. 2018), the present study identified a peri‐implantitis incidence of 7.6% at the implant level, slightly lower than rates reported previously (Diaz et al. 2022; C.‐T. Lee et al. 2017; dos Reis et al. 2025).

Several studies have shown that patients with a history of periodontitis are at an elevated risk of developing peri‐implantitis compared to those without periodontitis history (Berglundh et al. 2018; Dreyer et al. 2018; Galarraga‐Vinueza et al. 2025; Sgolastra et al. 2015). In contrast, less evidence is available regarding the role of current periodontitis status (Galarraga‐Vinueza et al. 2025). This distinction is clinically relevant because the term “history of periodontitis” encompasses patients with markedly different periodontal conditions, ranging from successfully treated and stable disease to ongoing inflammation (Chapple et al. 2018). Incorporating current periodontitis status, rather than relying solely on a history of periodontitis, may allow for a more accurate assessment of peri‐implantitis risk, as ongoing periodontal infection may facilitate the persistence and transmission of pathogenic microorganisms to peri‐implant tissues. Among all evaluated predictors, periodontitis showed the most consistent association with peri‐implantitis. The increased risk remained evident across survival (Figure 3), multivariable (Table 3), robustness, and follow‐up–restricted sensitivity analyses, including cohorts with at least 3 and 5 years of follow‐up (Table 4 and Figure 4). This consistency strengthens the evidence supporting periodontitis, particularly when accompanied by persistent periodontal pockets and bleeding on probing, as a major risk indicator for peri‐implantitis. These findings are supported by a recent systematic review and meta‐analysis demonstrating that “active” periodontitis is associated with approximately fourfold greater odds of peri‐implantitis, despite heterogeneity in the definitions of active periodontitis across studies (Galarraga‐Vinueza et al. 2025). Similar findings have been reported in individual cohort studies (Cho‐Yan Lee et al. 2012; Pimentel et al. 2018; Vagia et al. 2021). Collectively, these findings highlight the importance of considering periodontal disease status, rather than history alone, when assessing peri‐implantitis risk.

Previous evidence has shown that implants with marginal bone loss during healing and early post‐loading phases tend to develop progressive peri‐implant bone loss (Galindo‐Moreno et al. 2015). In addition, marginal bone loss in the first year of functional loading markedly increases the odds of peri‐implantitis (Ravidà et al. 2023; Windael et al. 2021). In support of these findings, PLCBL was associated with peri‐implantitis in the primary analysis of the present study. Although the direction of the association was generally preserved across the additional robustness analyses, the magnitude of the effect was attenuated in the follow‐up–restricted cohorts, particularly among implants with at least 5 years of follow‐up. This attenuation may reflect the reduced sample size and number of events available in the restricted cohorts, resulting in less precise estimates. Alternatively, PLCBL may exert its greatest influence during the early stages of peri‐implant disease development. Although the pathways linking marginal bone loss to peri‐implantitis were not directly examined in this study, PLCBL may create sheltered peri‐implant niches and non–bone‐contact surfaces that facilitate biofilm colonization and promote the development of peri‐implant disease (Albrektsson et al. 2017). Further studies are needed to clarify the long‐term role of PLCBL in peri‐implantitis progression.

In this cohort, residual cement was detected in approximately 13% of cement‐retained restorations. Excess cement was associated with lower peri‐implantitis‐free survival (Figure 3) and an increased risk of peri‐implantitis (Table 3). The association remained positive across the robustness analyses, although its magnitude was attenuated in the follow‐up–restricted cohorts. This attenuation may reflect the reduced number of implants and events available after applying stricter follow‐up criteria, resulting in less precise estimates. Although the present study was not designed to evaluate temporal effects of excess cement, it is possible that excess cement exerts a greater influence during the earlier stages of peri‐implant disease development. The observed association is consistent with previous clinical studies identifying residual cement as a risk indicator for peri‐implant disease (Korsch et al. 2014, 2015; Linkevicius et al. 2013; Staubli et al. 2017; Wilson Jr. 2009). The detrimental effect of residual cement is likely related to its plaque‐retentive surface and the limited accessibility of the submucosal area for adequate cleaning. Furthermore, inflammatory signs around affected implants often improve or resolve following cement removal, supporting a direct role of residual cement in peri‐implant tissue inflammation (Korsch et al. 2014, 2015; Staubli et al. 2017; Wilson Jr. 2009). Taken together, these findings support residual cement as a potentially modifiable risk factor for peri‐implantitis and emphasize the importance of meticulous cementation protocols and effective removal of excess cement during prosthesis delivery.

Hypothyroidism and previous bone grafting did not remain in the final model but demonstrated significant associations with peri‐implantitis in bivariate and survival analyses. Although a previous retrospective study found no significant association between hypothyroidism and peri‐implantitis (Dalago et al. 2017), growing evidence links hypothyroidism to increased prevalence and severity of periodontitis, possibly through alterations in bone metabolism, inflammatory responses, and oral microbiome dysbiosis (AlAhmari et al. 2024; Inchingolo et al. 2024; Puthiyapurayil et al. 2025; Song et al. 2021). These observations highlight the need for further investigation into a potential link between hypothyroidism and peri‐implantitis. Systematic reviews have not identified a clear difference in peri‐implantitis risk between implants placed in augmented and non‐augmented sites (Salvi et al. 2018; Sanz‐Sánchez et al. 2018); however, the available evidence is limited by heterogeneous study designs and the pooling of different augmentation procedures. With respect to sinus elevation, a cross‐sectional study reported a significant association between lateral‐window sinus floor elevation and peri‐implantitis (Stacchi et al. 2021), following a similar trend to that observed in the present study. Overall, direct evidence linking specific bone augmentation procedures to peri‐implantitis remains scarce, underlining the need for further well‐designed studies.

### 4.4 Strengths and Limitations

A major strength of this study is the comprehensive evaluation of multiple patient, implant/site‐, and prosthesis‐related factors, a few of which have been poorly explored. In addition, the use of time‐to‐event analyses allowed consideration of both the occurrence and timing of implant complications. This approach provided additional insight into implant failure and peri‐implantitis by accounting for differences in follow‐up duration among implants.

The limitations of this study should be acknowledged. Its retrospective design introduces the possibility of selection bias, residual confounding, and exposure misclassification due to reliance on self‐reported information. In addition, although the overall sample size was representative, the relatively low number of events for some outcomes limited the number of variables that could be retained in the multivariable models and may have contributed to imprecision and inflation of some effect estimates. To address this limitation, several complementary analyses, including clustered bootstrap resampling, penalized regression methods, and sensitivity analyses, were performed. These generally supported the direction of the identified associations, although with varying degrees of robustness. Overall, the findings across models should be considered exploratory and hypothesis‐generating and require confirmation in larger prospective cohorts, although some associations, particularly that between periodontitis and peri‐implantitis, showed greater consistency across analyses.

The application of strict predefined eligibility and exclusion criteria, together with the use of a complete‐case approach, may also have influenced the composition of the final analytical cohort and limited the generalizability of the findings. Variability in radiographic acquisition should be considered, as strict standardization could not be confirmed for all implants. However, radiographs of insufficient quality were excluded, and peri‐implant bone‐level measurements were performed by a trained and calibrated examiner with excellent reproducibility. Finally, despite the broad range of variables evaluated, some potentially relevant factors, such as peri‐implant soft‐tissue phenotype, could not be assessed because of incomplete chart data. In addition, the evaluation of prosthetic variables was constrained by the retrospective design. Radiographic assessment may underestimate the presence of residual cement and does not allow complete three‐dimensional evaluation of restoration contours and adaptation, particularly at the buccal and lingual aspects. Therefore, causal inferences remain limited, and well‐designed prospective longitudinal studies with adequate follow‐up are needed to validate these findings and further elucidate the factors influencing peri‐implant outcomes.

## 5 Conclusion

Self‐reported penicillin allergy and anterior implant location were associated with early implant failure, whereas the use of antihypertensive medications was related to improved early implant survival. Hypertension, implant placement in previously augmented sites, bone‐level platform placement, and implant manufacturer emerged as predictors of late implant failure. Periodontitis, excess cement, and PLCBL were associated with peri‐implantitis, with periodontitis showing the most consistent association across analyses. Overall, the findings of this study should be considered exploratory and hypothesis‐generating and warrant confirmation in larger prospective studies. Clinically, several identified factors may represent modifiable targets for improving implant outcomes.

## Author Contributions

**Nathalia Vilela:** writing – original draft, writing – review and editing, investigation, data curation. **Bruno C. V. Gurgel:** data curation, investigation, writing – original draft, writing – review and editing. **Hélio D. P. da Silva:** formal analysis. **Claudio M. Pannuti:** writing – review and editing. **Poliana M. Duarte:** conceptualization, methodology, investigation, supervision, project administration, writing – original draft, writing – review and editing.

## Funding

This work was supported by Coordenação de Aperfeiçoamento de Pessoal de Nível Superior, Finance Code 001.

## Ethics Statement

The study was approved by the University of Florida Institutional Review Board (IRB202101382) and conducted in accordance with the observational studies in epidemiology (STROBE) guidelines. This study was approved as exempt because it poses minimal risk and was approved under the following exempt category/categories: (4) (iii) Secondary research for which consent is not required.

## Conflicts of Interest

The authors declare no conflicts of interest.

## Supporting information


**Data S1:** clr70181‐sup‐0001‐DataS1.zip.

## Data Availability

The data that support the findings of this study are available from the corresponding author upon reasonable request.
