# Supplementary material for: Risk Factors for Implant Failure and Peri‐Implantitis: A Retrospective Cohort Study
Source: Clin Oral Implants Res. 2026 Sep 25;37(11):1386–401. doi: 10.1111/clr.70181 (PMC13743788; doi:10.1111/clr.70181)
Supplement: Supplementary file 1 [file CLR-37-1386-s001.zip › clr70181-sup-0006-Tables.docx]

| **Table S1-** Distribution and frequency of implants according to patient-related variables and the occurrence of early implant failure, late implant failure, and peri-implantitis. | | | | | | | | | | | | | | |
| --- | --- | --- | --- | --- | --- | --- | --- | --- | --- | --- | --- | --- | --- | --- |
| **Variables** | **Early failure** | | | |  | **Late failure** | | | |  | **Peri-implantitis** | | | |
|  | No | | Yes | |  | No | | Yes | |  | No | | Yes | |
|  | n | % | n | % |  | n | % | n | % |  | n | % | n | % |
| **Sex** |  |  |  |  |  |  |  |  |  |  |  |  |  |  |
| Male | 252 | 47.1 | 9 | 52.6 |  | 244 | 47.2 | 8 | 44.4 |  | 232 | 47.1 | 19 | 46.3 |
| Female | 283 | 52.9 | 10 | 47.4 |  | 273 | 52.8 | 10 | 55.6 |  | 261 | 52.9 | 22 | 53.7 |
| **Smoking** |  |  |  |  |  |  |  |  |  |  |  |  |  |  |
| Current | 71 | 13.3 | 0^†^ | 0 |  | 71 | 13.7 | 0^†^ | 0.0 |  | 69 | 14.0 | 2 | 4.9 |
| Former | 91 | 17.0 | 6 | 31.6 |  | 87 | 16.8 | 4 | 22.2 |  | 80 | 16.2 | 11 | 26.8 |
| Never | 373 | 69.7 | 13 | 68.4 |  | 359 | 69.5 | 14 | 77.8 |  | 344 | 69.8 | 28 | 68.3 |
| **Daily alcohol consumption** |  |  |  |  |  |  |  |  |  |  |  |  |  |  |
| Yes | 42 | 7.9 | 3 | 15.8 |  | 39 | 7.5 | 3 | 16.7 |  | 38 | 7.7 | 4 | 9.8 |
| No | 493 | 92.1 | 16 | 84.2 |  | 478 | 92.5 | 15 | 83.3 |  | 455 | 92.3 | 37 | 90.2 |
| **Hypertension** |  |  |  |  |  |  |  |  |  |  |  |  |  |  |
| Yes | 139 | 30.0 | 4 | 21.1 |  | 129 | 25.0 | 10 | 55.6^*^ |  | 126 | 25.6 | 13 | 31.7 |
| No | 396 | 70.0 | 15 | 78.9 |  | 388 | 75.0 | 8 | 44.4^*^ |  | 367 | 74.4 | 28 | 68.3 |
| **Diabetes** |  |  |  |  |  |  |  |  |  |  |  |  |  |  |
| Yes | 58 | 10.8 | 0^†^ | 0 |  | 58 | 11.2 | 0^†^ | 0.0 |  | 439 | 89.0 | 37 | 90.2 |
| No | 477 | 89.2 | 19 | 100 |  | 459 | 88.8 | 18 | 100 |  | 54 | 11.0 | 4 | 9.8 |
| **Hypothyroidism** |  |  |  |  |  |  |  |  |  |  |  |  |  |  |
| Yes | 56 | 10.5 | 4 | 21.1 |  | 54 | 10.4 | 2 | 11.1 |  | 45 | 9.1 | 11 | 26.8^*^ |
| No | 479 | 89.5 | 15 | 78.9 |  | 463 | 89.6 | 16 | 88.9 |  | 448 | 90.9 | 30 | 73.2^*^ |
| **Osteoporosis** |  |  |  |  |  |  |  |  |  |  |  |  |  |  |
| Yes | 34 | 6.4 | 1 | 5.3 |  | 34 | 6.6 | 0^†^ | 0.0 |  | 34 | 6.9 | 0^†^ | 0.0 |
| No | 501 | 93.6 | 18 | 94.7 |  | 483 | 93.4 | 18 | 100 |  | 459 | 93.1 | 41 | 100 |
| **Self-reported penicillin allergy** |  |  |  |  |  |  |  |  |  |  |  |  |  |  |
| Yes | 39 | 7.3 | 7 | 36.8^*^ |  | 35 | 6.8 | 4 | 22.2^*^ |  | 34 | 6.9 | 5 | 12.2 |
| No | 496 | 92.7 | 12 | 63.2^*^ |  | 482 | 93.2 | 14 | 77.8^*^ |  | 459 | 93.1 | 36 | 87.8 |
| **Antihypertensive medications** |  |  |  |  |  |  |  |  |  |  |  |  |  |  |
| Yes | 234 | 43.7 | 4 | 21.1^*^ |  | 222 | 42.9 | 12 | 66.7 |  | 212 | 43.0 | 22 | 53.7 |
| No | 301 | 56.3 | 15 | 78.9^*^ |  | 295 | 57.1 | 6 | 33.3 |  | 281 | 57.0 | 19 | 46.3 |
| **Anti-inflammatory medications** |  |  |  |  |  |  |  |  |  |  |  |  |  |  |
| Yes | 174 | 32.5 | 8 | 42.1 |  | 166 | 32.1 | 8 | 44.4 |  | 158 | 32.0 | 16 | 39.0 |
| No | 361 | 67.5 | 11 | 57.9 |  | 351 | 67.9 | 10 | 55.6 |  | 335 | 68.0 | 25 | 61.0 |
| **Antidepressant medications** |  |  |  |  |  |  |  |  |  |  |  |  |  |  |
| Yes | 100 | 18.7 | 5 | 26.3 |  | 94 | 18.2 | 6 | 33.3 |  | 92 | 18.7 | 8 | 19.5 |
| No | 435 | 81.3 | 14 | 73.7 |  | 423 | 81.8 | 12 | 66.7 |  | 401 | 81.3 | 33 | 80.5 |
| **Antiresorptive medications** |  |  |  |  |  |  |  |  |  |  |  |  |  |  |
| Yes | 43 | 8 | 2 | 10.5 |  | 43 | 8.3 | 0^†^ | 0.0 |  | 39 | 7.9 | 4 | 9.8 |
| No | 492 | 92 | 17 | 89.5 |  | 474 | 91.7 | 18 | 100 |  | 454 | 92.1 | 37 | 90.2 |
| **History of periodontitis** |  |  |  |  |  |  |  |  |  |  |  |  |  |  |
| Yes | 437 | 81.7 | 18 | 94.7 |  | 420 | 81.2 | 17 | 94.4 |  | 397 | 80.5 | 39 | 95.1^*^ |
| No | 98 | 18.3 | 1 | 5.3 |  | 97 | 18.8 | 1 | 5.6 |  | 96 | 19.5 | 2 | 4.9^*^ |
| **Periodontitis** |  |  |  |  |  |  |  |  |  |  |  |  |  |  |
| Active | 344 | 64.3 | 9 | 52.6 |  | 185 | 35.8 | 6 | 33.3 |  | 166 | 33.7 | 25 | 61.0^*^ |
| Non-active | 191 | 35.7 | 10 | 47.4 |  | 332 | 64.2 | 12 | 66.7 |  | 327 | 66.3 | 16 | 39.0^*^ |
| **SPT frequency** |  |  |  |  |  |  |  |  |  |  |  |  |  |  |
| ≤ 1 time/ year | 196 | 36.6 | 12 | 63.2 |  | 209 | 40.4 | 6 | 33.3 |  | 198 | 40.2 | 16 | 39 |
| 2-3 times/ year | 157 | 29.3 | 3 | 15.8 |  | 263 | 50.9 | 6 | 33.3 |  | 254 | 51.5 | 17 | 41.5 |
| ≥ 3 times/year | 182 | 34.1 | 4 | 21 |  | 45 | 8.7 | 6 | 33.3 |  | 41 | 8.3 | 8 | 19.5 |

* Statistically significantly different in the same row at p< 0.05 in the two-sided test of equality for column proportions. Tests assume equal variances and are adjusted for all pairwise comparisons within a row of each innermost subtable using the Bonferroni correction.

† This category is not used in comparisons because its column proportion is equal to zero or one.

| **Table S2 -** Distribution and frequency of implants according to implant/site-related variables and the occurrence of early implant failure, late implant failure, and peri-implantitis. | | | | | | | | | | | | | | |  |
| --- | --- | --- | --- | --- | --- | --- | --- | --- | --- | --- | --- | --- | --- | --- | --- |
| **Variables** | **Early failure** | | | |  | **Late failure** | | | |  | **Peri-implantitis** | | | | |
|  | No | | Yes | |  | No | | Yes | |  | No | | Yes | | |
|  | n | % | n | % |  | n | % | n | % |  | n | % | n | % | |
| **Implant location** |  |  |  |  |  |  |  |  |  |  |  |  |  |  | |
| Maxilla | 277 | 51.8 | 11 | 57.9 |  | 267 | 51.6 | 10 | 55.6 |  | 249 | 50.5 | 28 | 68.3^*^ | |
| Mandible | 258 | 48.2 | 8 | 42.1 |  | 250 | 48.4 | 8 | 44.4 |  | 244 | 49.5 | 13 | 31.7^*^ | |
| **Implant location** |  |  |  |  |  |  |  |  |  |  |  |  |  |  | |
| Posterior | 443 | 82.8 | 8 | 42.1^*^ |  | 431 | 83.4 | 12 | 66.7 |  | 413 | 83.8 | 29 | 70.7 | |
| Anterior | 92 | 17.2 | 11 | 57.9^*^ |  | 86 | 16.6 | 6 | 33.3 |  | 80 | 16.2 | 12 | 29.3 | |
| **Implant manufacturer** |  |  |  |  |  |  |  |  |  |  |  |  |  |  | |
| Straumann | 212 | 39.6 | 2 | 10.5^*^ |  | 210 | 40.6 | 2 | 11.1^*^ |  | 199 | 40.4 | 13 | 31.7 | |
| Astra | 259 | 48.4 | 9 | 47.4 |  | 249 | 48.2 | 10 | 55.6^*^ |  | 239 | 48.5 | 20 | 48.8 | |
| Zimmer | 41 | 7.7 | 2 | 10.5 |  | 38 | 7.4 | 3 | 16.7 |  | 35 | 7.1 | 5 | 12.2 | |
| Other | 23 | 4.3 | 6 | 31.6^*^ |  | 20 | 3.8 | 3 | 16.7 |  | 20 | 4 | 3 | 7.3 | |
| **Diameter** |  |  |  |  |  |  |  |  |  |  |  |  |  |  | |
| Narrow | 144 | 26.9 | 9 | 47.4^*^ |  | 137 | 26.5 | 7 | 38.9 |  | 131 | 26.5 | 13 | 31.7 | |
| Regular | 253 | 47.3 | 10 | 52.6 |  | 246 | 47.6 | 7 | 38.9 |  | 232 | 47.1 | 21 | 51.2 | |
| Wide | 138 | 25.8 | 0^†^ | 0^†^ |  | 134 | 25.9 | 4 | 22.2 |  | 130 | 26.4 | 7 | 17.1 | |
| **Length** |  |  |  |  |  |  |  |  |  |  |  |  |  |  | |
| Short (≤ 8 mm) | 59 | 11.0 | 1 | 5.3 |  | 57 | 10.5 | 2 | 11.1 |  | 56 | 11.4 | 3 | 7.3 | |
| Conventional (> 8mm) | 476 | 89.0 | 18 | 94.7 |  | 460 | 89.5 | 16 | 88.9 |  | 437 | 88.6 | 38 | 92.7 | |
| **Implant platform-abutment interface** |  |  |  |  |  |  |  |  |  |  |  |  |  |  | |
| Bone-level | 403 | 75.3 | 18 | 94.7^*^ |  | 386 | 74.7 | 17 | 94.4^*^ |  | 367 | 74.4 | 35 | 85.4 | |
| Tissue-level | 132 | 24.7 | 1 | 5.3^*^ |  | 131 | 25.3 | 1 | 5.6^*^ |  | 126 | 25.6 | 6 | 14.6 | |
| **Healing mode** |  |  |  |  |  |  |  |  |  |  |  |  |  |  | |
| Submerged (two-stage) | 180 | 33.6 | 9 | 47.4 |  | 177 | 34.2 | 3 | 16.7 |  | 162 | 32.9 | 18 | 43.9 | |
| Non-submerged (one-stage) | 355 | 66.4 | 10 | 52.6 |  | 340 | 65.8 | 15 | 83.3 |  | 331 | 67.1 | 23 | 56.1 | |
| **Timing of placement** |  |  |  |  |  |  |  |  |  |  |  |  |  |  | |
| Immediate | 71 | 13.3 | 4 | 21.0 |  | 69 | 13.3 | 2 | 11.1 |  | 68 | 13.8 | 2 | 4.9 | |
| Delayed | 464 | 86.7 | 15 | 79.0 |  | 448 | 86.7 | 16 | 88.9 |  | 425 | 86.2 | 39 | 95.1 | |
| **Implant-tooth and implant-implant distance** |  |  |  |  |  |  |  |  |  |  |  |  |  |  | |
| Inadequate (< 1.5 / < 3 mm) | 153 | 28.6 | 7 | 36.8 |  | 145 | 28.0 | 8 | 44.4 |  | 355 | 72.0 | 27 | 65.9 | |
| Adequate (≥ 1.5 / ≥ 3 mm) | 382 | 71.4 | 12 | 63.2 |  | 372 | 72.0 | 10 | 55.6 |  | 138 | 28.0 | 14 | 34.1 | |
| **Insertion depth** |  |  |  |  |  |  |  |  |  |  |  |  |  |  | |
| Equicrestal | 266 | 49.7 | 6 | 31.6 |  | 258 | 49.9 | 8 | 44.4 |  | 245 | 49.7 | 20 | 48.8 | |
| Subcrestal | 207 | 38.7 | 10 | 52.6 |  | 201 | 38.9 | 6 | 33.3 |  | 192 | 38.9 | 15 | 36.6 | |
| Supracrestal | 62 | 11.6 | 3 | 15.8 |  | 58 | 11.2 | 4 | 22.2 |  | 56 | 11.4 | 6 | 14.6 | |
| **Operator** |  |  |  |  |  |  |  |  |  |  |  |  |  |  | |
| Faculty | 470 | 87.9 | 17 | 89.5 |  | 454 | 87.8 | 16 | 88.9 |  | 444 | 90.1 | 25 | 61.0^*^ | |
| Resident | 65 | 12.1 | 2 | 10.5 |  | 63 | 12.2 | 2 | 11.1 |  | 49 | 9.9 | 16 | 39.0^*^ | |
| **Bone grafting during implant placement** |  |  |  |  |  |  |  |  |  |  |  |  |  |  | |
| No | 389 | 72.7 | 7 | 36.8^*^ |  | 376 | 72.7 | 13 | 72.2 |  | 357 | 72.4 | 32 | 78.0 | |
| Yes | 146 | 27.3 | 12 | 63.2^*^ |  | 141 | 27.3 | 5 | 27.8 |  | 136 | 27.6 | 9 | 22.0 | |
| **Previous bone grafting** |  |  |  |  |  |  |  |  |  |  |  |  |  |  | |
| No | 267 | 49.9 | 12 | 63.2 |  | 261 | 50.5 | 6 | 33.3 |  | 251 | 50.9 | 15 | 36.6 | |
| Ridge preservation | 218 | 40.7 | 5 | 26.3 |  | 212 | 41.0 | 6 | 33.3 |  | 205 | 41.6 | 13 | 31.7 | |
| Guided bone regeneration | 31 | 5.8 | 2 | 10.5 |  | 27 | 5.2 | 4 | 22.2^*^ |  | 23 | 4.7 | 8 | 19.5^*^ | |
| Sinus lift | 19 | 3.6 | 0^†^ | 0^†^ |  | 17 | 3.3 | 2 | 11.1 |  | 14 | 2.8 | 5 | 12.2^*^ | |
| **Immediate provisionalization** |  |  |  |  |  |  |  |  |  |  |  |  |  |  | |
| No | 367 | 68.6 | 8 | 42.1^*^ |  | 162 | 31.3 | 6 | 33.3 |  | 150 | 30.4 | 18 | 43.9 | |
| Yes | 168 | 31.4 | 11 | 57.9^*^ |  | 355 | 68.7 | 12 | 66.7 |  | 343 | 69.6 | 23 | 56.1 | |
|  |  |  |  |  |  |  |  |  |  |  |  |  |  |  | |

* Statistically significantly different in the same row at p< 0.05 in the two-sided test of equality for column proportions. Tests assume equal variances and are adjusted for all pairwise comparisons within a row of each innermost subtable using the Bonferroni correction.

† This category is not used in comparisons because its column proportion is equal to zero or one.

| **Table S3 -** Distribution and frequency of implants according to prosthesis-related variables and the occurrence of late implant failure and peri-implantitis. | | | | | | | | | |
| --- | --- | --- | --- | --- | --- | --- | --- | --- | --- |
| **Variables** | **Late failure** | | | |  | **Peri-implantitis** | | | |
|  | No | | Yes | |  | No | | Yes | |
|  | n | % | n | % |  | n | % | n | % |
| **Type of restoration** |  |  |  |  |  |  |  |  |  |
| Single | 392 | 75.8 | 12 | 66.7 |  | 376 | 76.4 | 27 | 65.9 |
| Bridge/Splinted | 125 | 24.2 | 6 | 33.3 |  | 117 | 23.6 | 14 | 34.1 |
| **Crown retention** |  |  |  |  |  |  |  |  |  |
| Screw-retained | 227 | 43.9 | 8 | 44.4 |  | 215 | 43.6 | 20 | 48.8 |
| Cemented | 290 | 56.1 | 10 | 55.6 |  | 278 | 56.4 | 21 | 51.2 |
| **Crown contour** |  |  |  |  |  |  |  |  |  |
| Adequate | 431 | 83.4 | 17 | 94.4 |  | 414 | 84.0 | 33 | 80.5 |
| Overcontoured | 86 | 16.6 | 1 | 5.6 |  | 79 | 16.0 | 8 | 19.5 |
| **Marginal adaptation** |  |  |  |  |  |  |  |  |  |
| Adequate | 408 | 78.9 | 16 | 88.9 |  | 389 | 78.9 | 34 | 82.9 |
| Defective | 109 | 21.1 | 2 | 11.1 |  | 104 | 21.1 | 7 | 17.1 |
| **Interproximal contact** |  |  |  |  |  |  |  |  |  |
| Adequate | 433 | 83.8 | 14 | 77.8 |  | 416 | 84.4 | 30 | 73.2 |
| Open contact | 84 | 16.2 | 4 | 22.2 |  | 77 | 15.6 | 11 | 26.8 |
| **Excess cement** |  |  |  |  |  |  |  |  |  |
| Present | 37 | 7.2 | 3 | 16.7 |  | 31 | 6.3 | 9 | 22.0^*^ |
| Absent | 480 | 92.8 | 15 | 83.3 |  | 462 | 93.7 | 32 | 78.0^*^ |
| **Technical issues** |  |  |  |  |  |  |  |  |  |
| None | 480 | 92.8 | 14 | 77.8^*^ |  | 457 | 92.7 | 36 | 87.8 |
| Chipping | 6 | 1.2 | 0^†^ | 0.0 |  | 6 | 1.2 | 0 | 0.0 |
| Decementation | 10 | 1.9 | 0^†^ | 0.0 |  | 9 | 1.8 | 1 | 2.4 |
| Screw loosening | 18 | 3.5 | 4 | 22.2^*^ |  | 18 | 3.7 | 4 | 9.8 |
| Abutment loosening | 3 | 0.6 | 0^†^ | 0.0 |  | 3 | 0.6 | 0 | 0.0 |
| **Implant/prosthesis component fracture** |  |  |  |  |  |  |  |  |  |
| Yes | 6 | 1.2 | 4 | 22.2^*^ |  | 8 | 1.6 | 2 | 4.9 |
| No | 511 | 98.8 | 14 | 77.8^*^ |  | 485 | 98.4 | 39 | 95.1 |
| **Antagonist** |  |  |  |  |  |  |  |  |  |
| Natural teeth | 305 | 59.0 | 12 | 66.7 |  | 286 | 58.0 | 30 | 73.2 |
| Prosthesis | 212 | 41.0 | 6 | 33.3 |  | 207 | 42.0 | 11 | 26.8 |

* Statistically significantly different in the same row at p< 0.05 in the two-sided test of equality for column proportions. Tests assume equal variances and are adjusted for all pairwise comparisons within a row of each innermost subtable using the Bonferroni correction.

† This category is not used in comparisons because its column proportion is equal to zero or one.

| **Table S4 -** Distribution and frequency of implants according to biological variables and the occurrence of late implant failure and peri-implantitis. | | | | | | | | | |
| --- | --- | --- | --- | --- | --- | --- | --- | --- | --- |
| **Variables** | **Late failure** | | | |  | **Peri-implantitis** | | | |
|  | No | | Yes | |  | No | | Yes | |
|  | n | % | n | % |  | n | % | n | % |
| **Pre-loading crestal bone loss** |  |  |  |  |  |  |  |  |  |
| Yes | 118 | 22.8 | 9 | 50.0^*^ |  | 113 | 22.9 | 13 | 31.7^*^ |
| No | 399 | 77.2 | 9 | 50.0^*^ |  | 380 | 77.1 | 28 | 68.3^*^ |
| **Peri-implantitis** |  |  |  |  |  |  |  |  |  |
| Yes | 27 | 5.2 | 14 | 77.8^*^ |  |  |  |  |  |
| No | 490 | 94.8 | 4 | 22.2^*^ |  |  |  |  |  |

* Statistically significantly different in the same row at p< 0.05 in the two-sided test of equality for column proportions. Tests assume equal variances and are adjusted for all pairwise comparisons within a row of each innermost subtable using the Bonferroni correction.

**Table S5 -** Variable selection using penalized regression (LASSO).

| **Event** | **Predictor** | **Retained by primary mixed-effects logistic regression** | **Retained by LASSO** |
| --- | --- | --- | --- |
| Early implant failure | Allergy to penicillin | Yes | Yes |
| Early implant failure | Anterior implant location | Yes | Yes |
| Early implant failure | Implant manufacturer | No | Yes |
| Early implant failure | Antihypertensive medication use | Yes | No |

**Table S6 -** Assessment of multicollinearity using variance inflation factors (VIFs) for the final multivariable models.

| **Outcome** | **Predictors** | **VIF range** | **Interpretation** |  |
| --- | --- | --- | --- | --- |
| Early implant failure | Penicillin allergy, implant location, antihypertensive medication | 1.01–1.06 | No relevant collinearity | |
| Late implant failure | Hypertension, previous bone grafting, implant platform-abutment interface, implant manufacturer | 1.01–1.83 | Low collinearity | |
| Peri-implantitis | Periodontitis status, excess cement, PCBL | 1.00–1.01 | No relevant collinearity |  |

PLCBL: pre-loading crestal bone loss
