# Supplementary material for: Risk Factors for Implant Failure and Peri‐Implantitis: A Retrospective Cohort Study
Source: Clin Oral Implants Res. 2026 Sep 25;37(11):1386–401. doi: 10.1111/clr.70181 (PMC13743788; doi:10.1111/clr.70181)
Supplement: Supplementary file 1 [file CLR-37-1386-s001.zip › clr70181-sup-0007-Supinfo1.docx]

**Supplementary Results**

**Bivariate analyses**

Table S1 presents the bivariate comparisons between patient-related variables and the occurrence of early implant failure, late implant failure, and peri-implantitis at the implant level. A significantly higher proportion of early implant failures was observed among patients with a penicillin allergy, whereas a lower proportion of early failures was noted among those using antihypertensive medications, compared with their counterparts without these conditions (p < 0.05). Late implant failure occurred significantly more frequently among patients with hypertension and among those with a penicillin allergy compared with patients without these conditions (p < 0.05). Peri-implantitis was significantly more frequent among patients with hypothyroidism and among those with a history of periodontitis or current periodontitis compared with patients without these conditions (p < 0.05).

**Table S2** presents the bivariate comparisons between implant/site-related variables and the occurrence of early implant failure, late implant failure, and peri-implantitis. Early failures occurred more frequently in implants placed in the anterior region, in those with narrow diameters, in bone-level implants, in sites that received bone grafting during implant placement, and in immediately provisionalized implants (p < 0.05). Straumann implants showed a lower proportion of early failures, whereas implants classified as “other brands” presented a higher proportion of early failures (p < 0.05). A higher proportion of late failures was observed in Astra compared with Straumann implants, in bone-level implants compared with tissue-level implants, and in sites previously treated with GBR compared with ridge preservation or non-grafted sites (p < 0.05). Peri-implantitis occurred more frequently in implants placed in the maxilla, in cases treated by residents, and in sites that had undergone GBR or sinus lift procedures (p < 0.05).

Table S3 presents the bivariate comparisons between prosthesis-related variables and the occurrence of late implant failure and peri-implantitis. Late implant failure was significantly associated with technical complications (p < 0.05), occurring more frequently in cases presenting screw loosening or implant/prosthesis component fracture. Peri-implantitis occurred more frequently in implants where cement remnants were identified (p < 0.05).

**Table S4** presents the bivariate comparisons between biologic-related variables and the occurrence of late implant failure and peri-implantitis. Late implant failures occurred more frequently in implants that exhibited PLCBL and in those diagnosed with peri-implantitis (p < 0.05). Peri-implantitis occurred more frequently in implants that showed PLCBL (p < 0.05).

**Events-per-variable (EPV)**

The peri-implantitis model included 41 events and three final predictors (EPV = 13.7), exceeding the commonly recommended threshold of 10 events per variable. In contrast, the early implant failure (19 events; EPV = 6.3) and late implant failure (18 events; EPV = 6.0) models did not meet this criterion. Therefore, the peri-implantitis analyses were considered confirmatory, whereas the implant failure analyses should be interpreted as exploratory and hypothesis-generating.

**Penalized regression: Least absolute shrinkage and selection operator (LASSO) and Elastic Net**

The predictors retained by the primary mixed-effects logistic regression model and the LASSO analysis are presented in Table S5. Penicillin allergy and anterior implant location were retained by both methods, supporting the robustness of these associations for early implant failure. The third predictor differed between approaches, with implant manufacturer selected by LASSO and antihypertensive medication use retained in the primary model. These findings suggest overall consistency for the main predictors, while indicating some uncertainty in variable selection. Figure S2 shows the cross-validation results for the early implant failure LASSO model, and the selection of the optimal penalty parameter (λ).

For late implant failure, both LASSO and Elastic Net retained the implant platform-abutment interface variable. However, the penalized effect estimates (exp[β] = 1.46 and 1.61, respectively) were substantially smaller than the hazard ratio estimated in the primary Cox model, indicating attenuation of effect magnitude after penalization. Figure S4 shows the cross-validation results for the late implant failure LASSO model and the selection of the optimal penalty parameter (λ).

Because the peri-implantitis model met commonly recommended events-per-variable thresholds, LASSO was used primarily as an additional robustness assessment. The LASSO results were generally consistent with those obtained in the primary model, supporting the stability of the peri-implantitis findings. Figure S5 shows the cross-validation results for the peri-implantitis LASSO model and the selection of the optimal penalty parameter (λ).

**Multicollinearity**

Multicollinearity diagnostics indicated no evidence of relevant collinearity among predictors included in the final models. Variance inflation factors (VIF) were low across all analyses (range: 1.00–1.83) (Table S6).

Given the structural relationship between implant manufacturer and platform-abutment interface in the sample, an additional targeted collinearity diagnostic was performed for these two variables using generalized VIF. All Astra implants were bone-level, whereas Straumann implants included both bone-level and tissue-level designs. Both manufacturer and platform-abutment interface showed a VIF of 2.44, below commonly used thresholds for problematic multicollinearity (VIF > 5–10), suggesting that the observed relationship did not result in severe variance inflation within the fitted model. Nevertheless, given the structural overlap between these variables, their individual effects cannot be considered fully independent and should be interpreted with caution.

**Supplementary Figure Legends**

**Figure S1.** Comparison of odds ratios and 95% confidence intervals obtained from the primary mixed-effects logistic regression model and patient-level clustered bootstrap resampling (2,000 iterations) for early implant failure.

**Figure S2. Cross-validation results for the early implant failure LASSO model.**

**Figure S3.** Comparison of hazard ratios and 95% confidence intervals for late implant failure obtained from the primary mixed-effects Cox proportional hazards model, cluster-robust Cox proportional hazards regression, and patient-level clustered bootstrap resampling (2,000 iterations).

**Figure S4. Cross-validation results for the late implant failure LASSO model.**

**Figure S5. Cross-validation results for the peri-implantitis LASSO model.**
